# Expression and Function of the Endocannabinoid System in the Retina and the Visual Brain

**DOI:** 10.1155/2016/9247057

**Published:** 2015-12-29

**Authors:** Jean-François Bouchard, Christian Casanova, Bruno Cécyre, William John Redmond

**Affiliations:** ^1^Laboratoire de Neuropharmacologie, École d'optométrie, Université de Montréal, C.P. 6128 Succursale Centre-Ville, Montréal, QC, Canada H3C 3J7; ^2^Laboratoire des Neurosciences de la Vision, École d'optométrie, Université de Montréal, C.P. 6128 Succursale Centre-Ville, Montréal, QC, Canada H3C 3J7

## Abstract

Endocannabinoids are important retrograde modulators of synaptic transmission throughout the nervous system. Cannabinoid receptors are seven transmembrane G-protein coupled receptors favoring G_i/o_ protein. They are known to play an important role in various processes, including metabolic regulation, craving, pain, anxiety, and immune function. In the last decade, there has been a growing interest for endocannabinoids in the retina and their role in visual processing. The purpose of this review is to characterize the expression and physiological functions of the endocannabinoid system in the visual system, from the retina to the primary visual cortex, with a main interest regarding the retina, which is the best-described area in this system so far. It will show that the endocannabinoid system is widely present in the retina, mostly in the through pathway where it can modulate neurotransmitter release and ion channel activity, although some evidence also indicates possible mechanisms via amacrine, horizontal, and Müller cells. The presence of multiple endocannabinoid ligands, synthesizing and catabolizing enzymes, and receptors highlights various pharmacological targets for novel therapeutic application to retinal diseases.

## 1. Introduction

The endocannabinoid (eCB) system is a complex neuromodulatory system consisting of two classical receptors, cannabinoid receptor type 1 (CB1R) and cannabinoid receptor type 2 (CB2R), their endogenous ligands named endocannabinoids, and enzymes responsible for their synthesis and degradation. This system can modulate both inhibitory and excitatory synapses in a short- or long-lasting manner. They mostly act through a retrograde mechanism in which the postsynaptic on demand release of eCBs will lead to a presynaptic CB1R activation in order to reduce transmitter release [1–3]. These eCBs, the two best defined of which are* N-*arachidonoyl ethanolamide, also known as anandamide (AEA), and 2-arachidonoyl glycerol (2-AG), are then quickly degraded, mostly via fatty acid amide hydrolase (FAAH) activity for AEA and monoacylglycerol lipase (MAGL) for 2-AG. This retrograde release can also act on CB2R in large part situated on glial cells in the adult nervous system [2, 4]. CB1R and CB2R are both 7-transmembrane G-protein coupled receptors with a strong preference toward G_i/o_ coupling [5, 6], although G_q_ [7] and G_s_ [8] coupling has also been reported for CB1R in the past. This can lead, among others, to a reduction of presynaptic glutamate and GABA release. Cannabinoids could also have neuroprotective and plasticity-mediating properties. For general reviews on the eCB signaling system, see [2, 9–11].

In recent years, the role of eCBs in visual function has been intensely studied. Recent reports suggest that the eCB system could play an instrumental role at all levels of the visual system. The vast majority of these studies focused on the effects of eCBs on adult retinal functions and only a few investigated the effects of cannabinoids on visual perception.

Reports showed that Δ9-tetrahydrocannabinol (THC) increased the recovery time from bright foveal glare by several seconds [12]. Acute effects on vision from smoking marijuana include a reduction in Vernier and Snellen acuity, alterations in color discrimination, and an increase in photosensitivity [13, 14]. Some anecdotal reports also showed that Jamaican fishermen smoke marijuana to improve dim light vision when fishing at night [15]. These results have been corroborated by another study that measured more precisely the night vision of Moroccan fishermen using cannabis to improve visual perception [16]. The authors noted increased night vision following THC or cannabis ingestion and suggested that these effects were produced locally in the retina. These intriguing findings have certainly fascinated various researchers who then began to study the expression of the eCB system in the visual system with a focus on the adult retina. While still scarce, there are now increasing evidence of transient receptor and enzymatic machinery expression during development.  Section 2 and Section 3 of this review will describe the expression (Section 2) and function (Section 3) of eCBs in the adult retina, whilst Section 4 will focus on these during development (Section 4).

## 2. Cannabinoid Expression in the Visual System

### 2.1. Ocular Tissues

It is well established that marijuana consumption induces vasodilatation in conjunctiva noticeable by a reddish color change in the eyes and a reduction in intraocular pressure [12]. It was first believed that marijuana exerted its effects systemically through the central nervous system (CNS). However, it became evident that cannabinoids can act locally on cannabinoid receptors present in various cell types in the eye [17]. Physiological and biochemical studies demonstrated the presence of the eCB system in various regions of ocular tissues. The CB1R mRNA is found in the ciliary body of rat, bovine, and human as well as in the trabecular meshwork in bovine and human [18–21]. CB1R is also detected in the nonpigmented ciliary epithelium in human and bovine tissues and conjunctival epithelium in mouse and human [19, 22]. The bovine corneal epithelial cells express, among others, CB1R, MAGL, *α*/*β* hydrolase domain 6 (ABHD6), *α*/*β* hydrolase domain 12 (ABHD12), and N-acyl phosphatidylethanolamine phospholipase D (NAPE-PLD) mRNA [23]. Furthermore, AEA and 2-AG, as well as other* N*-acyl ethanolamides such as palmitoylethanolamide (PEA), are present in human ocular tissues, except for the lens [20, 24].

### 2.2. Adult Retina

#### 2.2.1. Endocannabinoids

The two major eCBs (AEA and 2-AG) are present in the retina of adult rodents [17], bovines [25], and humans [20]. Most of the studies comparing AEA to 2-AG expression in the retina concluded that 2-AG levels are significantly higher than AEA; thus human and bovine retinas contain 25 times more 2-AG compared to AEA [20, 25, 26]. PEA was detected in the rat and human and oleoylethanolamide (OEA), another endocannabinoid-like* N*-acyl ethanolamide, was also measured in the rat retina [17]. In human, 2-AG is mostly produced in the retina, while AEA and PEA are mainly expressed in the iris [20]. The endocannabinoid levels in the retina are presented in Table 1.

The content of eCBs varies in certain disease states, suggesting the importance of eCBs in maintaining ocular homeostasis. For instance, 2-AG levels decrease in the ciliary body of patients with glaucoma [20] but increase in the iris of patients with diabetic retinopathy [24]. Similarly, PEA levels are lower in the ciliary body and the choroid of glaucomatous human retinas [20] but higher in the ciliary body of diabetic retinopathy human retinas [24]. Patients with age-related macular degeneration (AMD) and diabetic retinopathy also showed widespread increases of AEA levels in the retina, choroid, ciliary body, and cornea [24]. It is believed that the upregulation of eCBs in these disorders may contribute to cell protection in part by the anti-inflammatory and neuroprotective properties of eCBs [24].

#### 2.2.2. Cannabinoid Receptors

**Figure 1:**
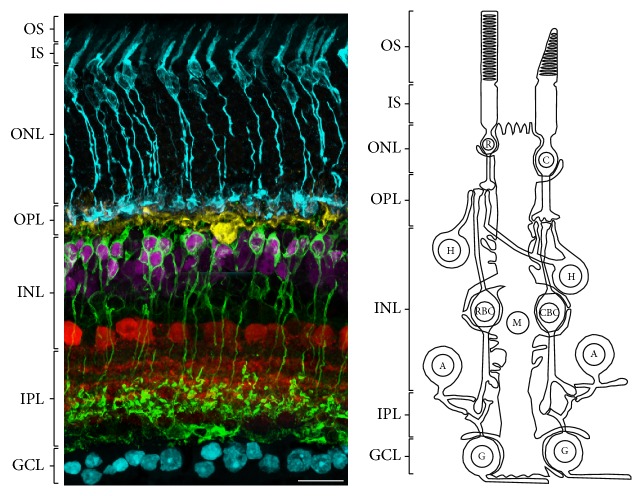
Schematic illustration representing the organization of the mouse retina. Rod (R) and cone (blue/C) photoreceptors have their cell bodies in the outer nuclear layer (ONL) and extend inner (IS) and outer (OS) segments. Photoreceptors axons synapse in the outer plexiform layer (OPL) with horizontal (yellow/H) and bipolar (magenta/RBC-CBC) cells. The inner nuclear layer (INL) also contains amacrine (red/A) and Müller cells (M). Bipolar cells synapse to amacrine and ganglion (blue/G) cells in the inner plexiform layer (IPL). Ganglion cell axons form the optic nerve in the ganglion cell layer (GCL) and carry signals to the brain.

**Figure 2:**
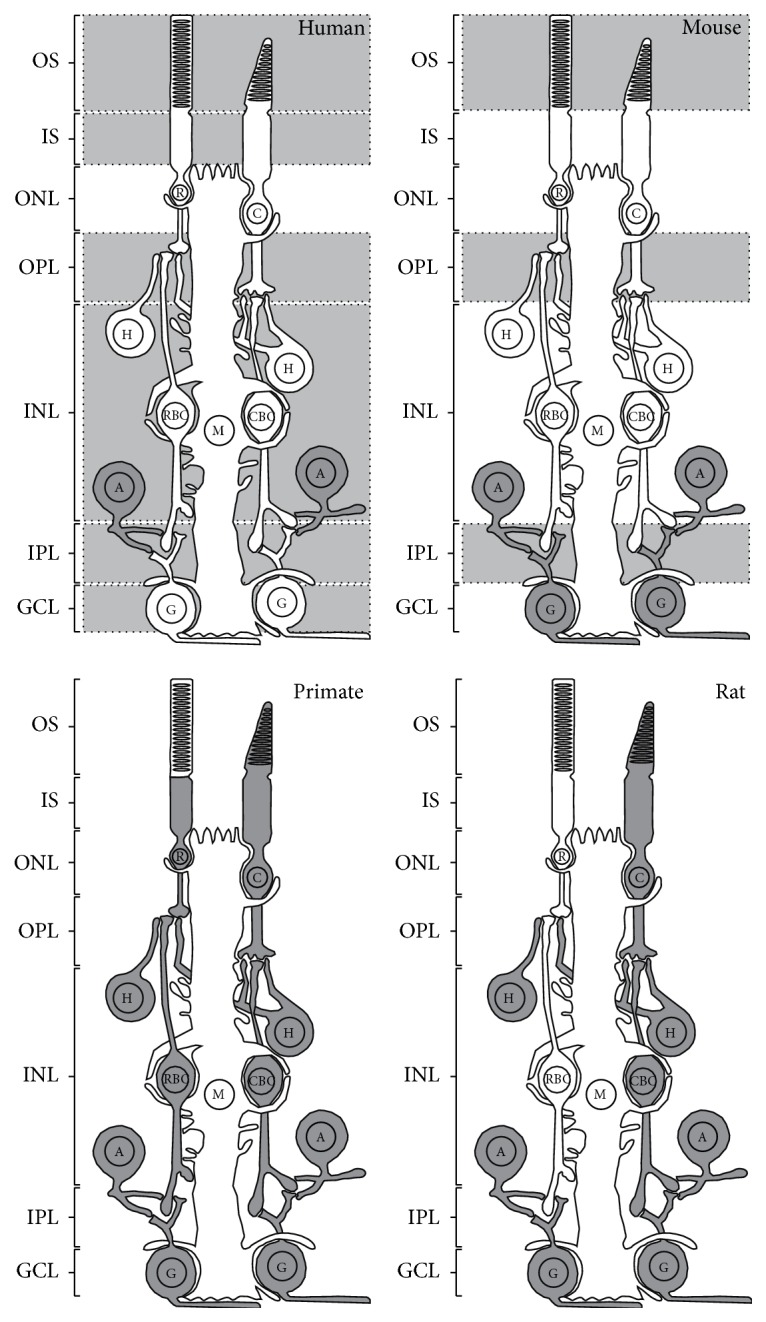
Schematic illustration representing the distribution of CB1R in the adult retina of several species. CB1R expression was demonstrated in dark gray retinal cells, while CB1R presence was noted in light gray retinal layers without precise localization. OS, outer segments of photoreceptors; IS, inner segments of photoreceptors; ONL, outer nuclear layer; OPL, outer plexiform layer; INL, inner nuclear layer; IPL, inner plexiform layer; GCL, ganglion cell layer; C, cones; R, rods; H, horizontal cells; CBC, cone bipolar cells; RBC, rod bipolar cells; A, amacrine cells; G, ganglion cells; M, Müller cells.

CB1R was extensively studied in the retina of various species using techniques such as* in situ* hybridization, reverse transcription polymerase chain reaction (RT-PCR), Western blot, or immunohistochemistry. CB1R was first localized in the ganglion cell layer (GCL) and inner nuclear layer (INL) of the rat retina [27]. Since then, CB1R expression was detected in the retinas of human, monkey, mouse, rat, chick, salamander, and goldfish with a similar labeling in the outer plexiform layer (OPL), inner plexiform layer (IPL), and GCL [17, 22]. A schematic illustration of the mouse retina organization is presented in Figure 1. Retinal protein distribution of CB1R is presented in Table 2 and its expression for different species is illustrated in Figure 2.

**Figure 3:**
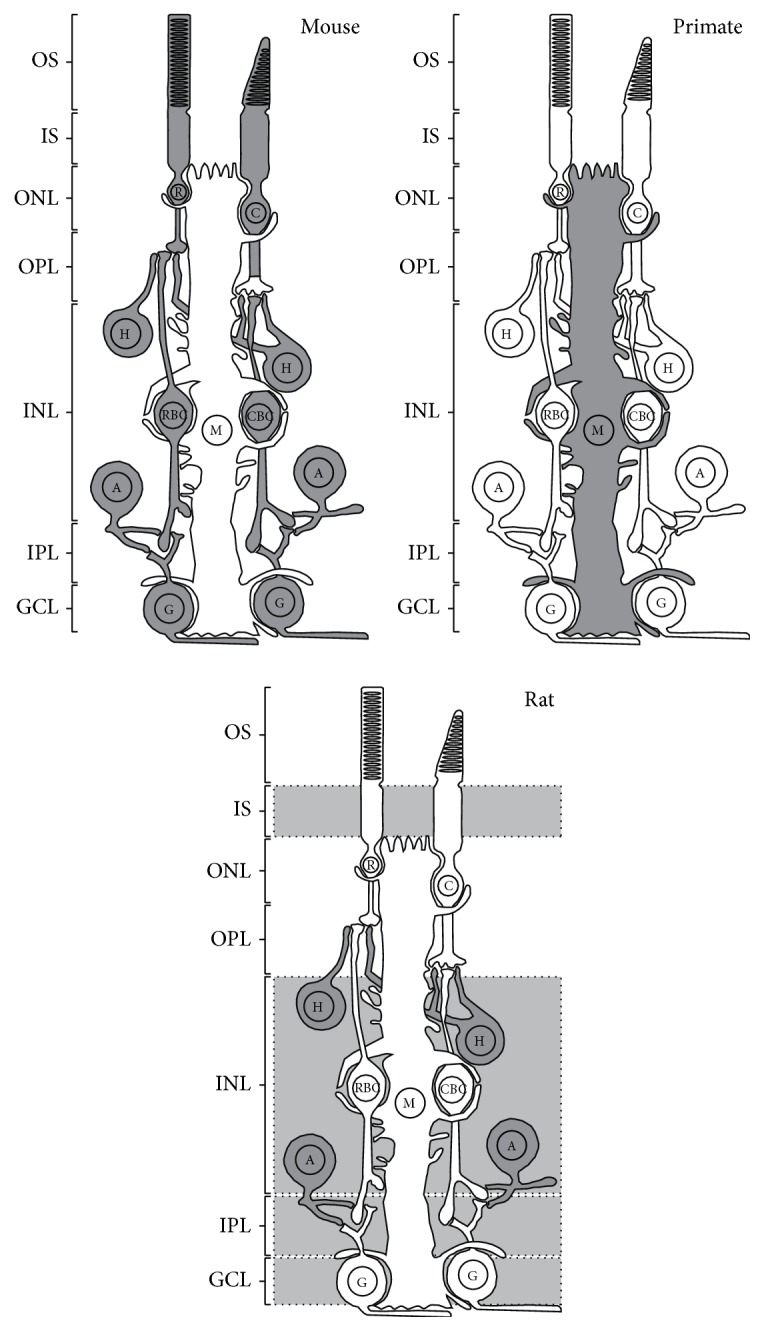
Schematic illustration showing the expression of CB2R in the adult retina of several species. CB1R expression was demonstrated in dark gray retinal cells, while CB2R presence was noted in light gray retinal layers without precise localization. OS, outer segments of photoreceptors; IS, inner segments of photoreceptors; ONL, outer nuclear layer; OPL, outer plexiform layer; INL, inner nuclear layer; IPL, inner plexiform layer; GCL, ganglion cell layer; C, cones; R, rods; H, horizontal cells; CBC, cone bipolar cells; RBC, rod bipolar cells; A, amacrine cells; G, ganglion cells; M, Müller cells. Scale bar: 20 *μ*m.

The cannabinoid receptor type 2 (CB2R) distribution in the retina has been less extensively studied than CB1R. This could be explained in part by the lack of specific markers for CB2R [28–30] and by initial reports revealing an expression pattern restricted to immune cells. It was first believed that CB2R was not expressed in the embryonic and adult rat retina [18, 27]. Then, CB2R mRNA localization was shown in photoreceptors, INL, and GCL of the rat retina [31]. More recently, protein expression of CB2R was detected in retinal pigment epithelium (RPE), inner segments of the photoreceptors, and horizontal and amacrine cells of the rat retina [32]. Furthermore, a recent report localized CB2R in cone and rod photoreceptors, horizontal cells, some amacrine cells, and bipolar and ganglion cells of the mouse retina [33]. CB2R is also expressed in the vervet monkey retina but exclusively in Müller cells [34]. Finally, CB2R mRNA expression was also recently found in the goldfish retina; however, its precise distribution is still unknown [35]. Retinal protein distribution of CB2R is presented in Table 3 and its expression for different species is illustrated in Figure 3.

#### 2.2.3. Cannabinoid-Like Receptors

In recent years, the G-protein coupled receptor 55 (GPR55) was suggested to act as a cannabinoid receptor since it interacts with AEA and THC [36]. Due to the lack of specificity of GPR55 markers, only one report studied its expression in the retina. In the vervet monkey retina, GPR55 is present exclusively in rods, with most prominent staining in their inner segments [37].

Another cannabinoid-like receptor present in the retina is the transient receptor potential vanilloid 1 (TRPV1), which binds eCBs such as AEA and N-arachidonoyl dopamine [38]. It was first localized in photoreceptor synaptic ribbons and in amacrine cells of goldfish and zebrafish retinas [39, 40]. In the rat retina, TRPV1 was found in microglial cells, blood vessels, and astrocytes and in neuronal structures such as synaptic boutons of both plexiform layers as well as in cell bodies of the INL and GCL [41]. TRPV1 mRNA was also detected in ganglion and Müller cells in the rat retina [42]. In the rabbit and human retina, TRPV1 is intensely expressed in the RPE [43]. TRPV1 is also present in the outer nuclear layer (ONL) and INL and at the end of the nerve fiber layer as well as in Müller cells of the rabbit retina [43].

The G-protein coupled receptor 18 (GPR18) is activated by N-arachidonoyl glycine (NAGly), the endogenous metabolite of AEA, and is suggested to be the “abnormal-CBD” (Abn-CBD) receptor [44]. Only one study examined the expression of GPR18 in the retina. It is mostly expressed in the IPL and OPL and in the endothelium of retinal vessels in the rat [45].

Furthermore, there is growing evidence that the intracellular peroxisome proliferator-activated receptors (PPARs) are the targets of cannabinoid ligands. PPARs belong to a family of nuclear receptors comprising three isoforms: *α*, *δ*, and *γ* (see [46] for review). PPAR *α*, *δ*, and *γ* are diffusely expressed in the retina and the RPE of humans and mice [47]. PPARs are expressed in cultures of primary RPE cells and ARPE-19 cells (a human RPE cell line) [48]. PPAR*γ*1 and PPAR*δ* are moderately expressed in both cell types, while PPAR*α* is only expressed in ARPE-19 cells. PPAR*α* and *δ* are also expressed in freshly isolated RPE, but PPAR*γ* is absent [48].

#### 2.2.4. Synthesizing and Catabolic Enzymes

The first retinal localization of the enzymes responsible for 2-AG synthesis was recently reported. Diacylglycerol lipase alpha (DAGL*α*) is present in the two synaptic layers of the mouse retina, namely, the OPL and the IPL [49]. Indeed, DAGL*α* is localized in postsynaptic terminals of type 1 OFF cone bipolar cells as well as in the dendrites of unidentified bipolar cells. DAGL*α* expression was recently found in the rat retina, as its presence was detected in cone and rod photoreceptors, horizontal cells' processes, some cone bipolar cells axonal connections, amacrine cells, and ganglion cells [50]. DAGL*β* is exclusively expressed in retinal blood vessels [49].

The hydrolyzing enzyme FAAH, which is responsible for the degradation of AEA, is localized in the retina of mice [49], rats [51, 52], and primates [53]. It was first detected in horizontal cells, dopamine amacrine cells, dendrites of starburst amacrine cells, and large ganglion cells of the rat retina [51]. Further studies revealed the expression of FAAH in cone photoreceptors, rod bipolar cells, and some ganglion cells in the rat retina as well as in the inner segments of photoreceptors, ONL, and GCL, in a subpopulation of amacrine and cone bipolar cells, and in the axon terminals of rod photoreceptors [49, 52]. In the vervet monkey retina, FAAH is localized in cones, cone pedicles, rod spherules, cone and rod bipolar cells, and ganglion cells [53].

The metabolizing enzyme MAGL is expressed in the mouse and rat retina. It was first detected in the OPL, IPL, and GCL [49]. In the IPL, MAGL is particularly present in two laminae: one in the central IPL and the other in the distal IPL. In the OPL, MAGL is found in rod spherules and cone pedicles. A recent study revealed that, in the rat retina, MAGL is expressed in amacrine and Müller cells as well as in the axonal connections of type 2 cone bipolar cells [50].

The expression of the metabolizing ABHD6, a serine hydrolase [54, 55], was reported in the mouse retina. It is localized in GABAergic amacrine cells and ganglion cells and in the dendrites of ganglion cells or displaced amacrine cells [49]. As for the synthesizing enzyme NAPE-PLD, a major synthesizing enzyme of AEA and OEA from lipids, it is expressed in the rat retina [56].

For now, no studies have revealed the presence of ABHD12 in the retina [55], as the study of ABHD12 requires the development of a specific antibody against this protein.

### 2.3. Cannabinoid System Expression in the Primary Visual Cortex

As CB1R is the most abundant GPCR in the CNS, it is not surprising that the eCB system is also present in several visual brain regions beyond the retina. For instance, CB1R is expressed throughout the dorsal lateral geniculate nucleus (dLGN) of vervet monkeys, with a prominent labeling in the magnocellular layers [57]. The catabolic enzyme FAAH shows the same pattern of expression, while the synthesizing enzyme NAPE-PLD is expressed homogenously throughout the dLGN [57]. All CB1R, FAAH, and NAPE-PLD are weakly expressed in the koniocellular layers. Furthermore, CB1R is expressed in the primary visual cortex (V1) of macaque monkeys, with the highest density observed in layers V-VI and the absence of labeling in layer IV [58]. CB1R is intensely expressed in layers II/III and VI of the striate cortex of the adult mouse [59], where it is mainly localized at vesicular GABA transporter-positive inhibitory nerve terminals. The eCB system also appears to be of importance in development as CB1R protein expression increased throughout the development of V1, with a specific laminar pattern of CB1R appearing at postnatal day 20 (P20) and remaining until adulthood [59].

## 3. Cannabinoid Function in the Visual System

### 3.1. Retina

In the retina, cannabinoids inhibit the release of various neurotransmitters. Indeed, CB1R agonists decrease the release of [^3^H]-noradrenaline and [^3^H]-dopamine in the guinea pig [60] via a G_i/o_-dependent mechanism [61, 62]. In general, though, the cannabinoid-mediated system in the vertebrate retina appears to act mainly via the “through” pathway, as cannabinoid receptors are concentrated in photoreceptors and bipolar and ganglion cells, that is, cells using glutamate as a principal neurotransmitter [63]. A first proof of concept for the importance of CB1R-mediated modulation of glutamate release came from the inhibition by cannabinoids of [^3^H]-D-aspartate (a substitute of L-glutamate for high-affinity uptake sites) release following ischemia or K^+^ channel activation in isolated bovine retina [64]. Some evidence also suggests a regulatory effect on GABA_A_ receptor-mediated inward currents from WIN55,212-2, a synthetic cannabinoid with similar affinity to both CB1R and CB2R, on low spontaneous transmission in embryonic chick amacrine cells [65]. It is important to note that, in this study, Warrier and Wilson used a high concentration of WIN55,212-2 which could produce off-target effects. At this concentration, the effect of WIN55,212-2 was not blocked by SR141716A, a selective CB1R inverse agonist. Due to the nature of WIN55,212-2, this effect could be mediated by CB2R via the activation of a TRP-family type of receptor or via other off-target mechanisms. Taken together, these various effects may suggest a possible cannabinoid-mediated tone in transmitter release. Yet, in* in vivo* electrophysiological studies, only CB2R, not CB1R, appears to cause changes to light responses in the mouse retina:* in vivo* recording of electroretinogram responses indicates that* cnr2*-KO animals exhibit increased a-wave amplitude under scotopic conditions and different light adaptation pattern in photopic conditions [33]. As this review aims at describing the anatomical distribution of cannabinoids in the retina, modulatory effects of cannabinoids will be reported by cell type.

#### 3.1.1. Epithelial Cells

In recent years, epithelial cells have been shown to be of importance in several models of pathology induced in cultured RPE cells. In one study, high glucose-mediated apoptosis in ARPE-19 RPE cells was reduced by the overexpression of FAAH, via CB1R blockade and via CB1R siRNA transfection, demonstrating a therapeutic potential for FAAH modulation in diabetic retinopathy [66]. An overexpression of CB1R and CB2R and downregulation of FAAH were also observed in response to oxidative stress in a model of AMD [67]. In a first study, cannabinoid agonists were shown to cause neuroprotection in these cells, although the specificity of these effects via CB1R or CB2R mechanism could not be made due to a lack of proper use of inhibitors or specific blockade of the receptors. However, in a follow-up study by the same authors, blockade of the overexpressed CB1R in this model via specific siRNA conveyed a neuroprotective effect by causing a downregulation of oxidative stress signaling and facilitating phosphatidylinositol 3′-kinase (PI_3 _K/AKT) activation, a fundamental signaling pathway for cellular apoptosis [68]. As the list of eCBs keeps expanding, so does the list of their potential targets. For example, the atypical endothelial cannabinoid receptor, CB_e_ (for review, see [69]), has recently shown promises as a possible inhibitor of vasoconstriction in the retinal microvasculature, an autoregulated CNS bed. Abn-CBD, an agonist of the atypical endothelial cannabinoid receptor, causes an inhibition of the retinal arteriole vasoconstriction induced by endothelin-1. This mechanism was independent of CB1R/CB2R as well as of GPR55, a receptor for which Abn-CBD is an agonist. N-arachidonoyl glycine, a putative GPR18 agonist, showed nearly identical effects as those of Abn-CBD. Both mechanisms took place via calcium-sensitive potassium channels (SKCa). The presence of the endothelium is important for both mechanisms, as these effects were highly reduced in its absence [45]. Abn-CBD via GPR18 also caused vasodilation of isolated perfused retinal arterioles, but only on precontracted vessels, hinting at a mechanism dependent on vascular tone. Intra- and extraluminal administration gave very different responses, and again the presence of epithelial cells was shown to be primordial in this mechanism [70, 71]. Various cannabinoids could activate PPAR*α* [72], although no studies have so far shown an effect of cannabinoids via PPAR*α* on the visual system. Interestingly, a recent study by Chen et al. has demonstrated that fenofibrate, a direct agonist of PPAR*α*, has a therapeutic potential for the treatment of diabetic retinopathy in models of type 1 diabetes [73]. It would be interesting to investigate if some of the effects of cannabinoids in this pathology could come from a similar mechanism.

#### 3.1.2. Photoreceptors

Different effects on rod and cone photoreceptors have been reported for the salamander and goldfish following WIN55,212-2 addition, with a potential biphasic response based on concentration for the goldfish. Delayed rectifier currents (*I*_K_) were suppressed by WIN55,212-2 in cones and rods, whereas Ca^2+^ currents (*I*_Ca_) were enhanced in rods and suppressed in cones [74, 75], which could potentially be translated to an increased transmitter release, thus reducing light sensitivity. A cannabinoid-mediated retrograde suppression of membrane currents via 2-AG release on goldfish cones in retinal slices was also tested by Fan and Yazulla [76]. These authors found the existence of a retrograde transmission in cones, with bipolar cell dendrites as the likely source of 2-AG. Furthermore, the retrograde suppression of *I*_K_ is mediated by Ca^2+^ dependent release of 2-AG from bipolar cell dendrites. Cannabinoids also preserved cone and rod structure, as well as function and synaptic connectivity with postsynaptic neurons in a transgenic model for autosomal dominant retinitis pigmentosa. Indeed, HU-210, a more potent and longer lasting synthetic analogue of THC, increased the scotopic a- and b-wave amplitudes in treated animals and preserved photoreceptor degeneration and synaptic contacts between photoreceptors and bipolar or horizontal cells [77].

#### 3.1.3. Bipolar Cells

As for photoreceptors, Straiker and Yazulla's groups were the first to describe a cannabinoid-mediated effect in the salamander and the goldfish, respectively [17]. WIN55,212-2 reversely inhibited *I*_Ca_ in the salamander [6]. On the goldfish large ON-type bipolar cells, cannabinoid agonists such as CP54490 (a full agonist at both CB1R and CB2R) and WIN55,212-2 inhibited *I*_K_, and this inhibition could be reversed by using SR141716A, an inverse agonist for CB1R [78]. As SR141716A did not cause by itself much increase in *I*_K_ of some ON bipolar cells and no increase on others, an eCB modulating tone present in this area remains unclear. This lack of direct modulation of bipolar cells by cannabinoids does not necessarily translate into no cannabinoid-mediated mechanism for these cells. Retrograde signaling from ganglion cells is still a plausible mechanism and was first demonstrated for 2-AG [76].

WIN55212-2 also inhibits the enhancement in *I*_K_ seen following the activation of D1 receptors. It has thus been proposed that the cannabinoid and dopaminergic system have opposite properties on bipolar cells. SR141716A and a pretreatment with* pertussis toxin* could both block this mechanism, although WIN55,212-2 by itself did not cause an increase in conductivity on *I*_K_ [78]. Cannabinoids effects on ON bipolar cells have thus been associated with a tonic effect, whereas D1-mediated effects have been described as phasic [63].

#### 3.1.4. Amacrine Cells

The neuroprotective effects of endogenous and synthetic cannabinoids on the viability of amacrine cells were studied using an* in vivo* AMPA excitotoxicity model of retinal neurodegeneration. AEA, HU-210, and methanandamide (a stable synthetic chiral analogue of AEA) afforded partial recovery following the AMPA-induced excitotoxicity of both bNOS-positive and cholinergic amacrine cells [79]. This neuroprotection is mediated by a mechanism involving CB1R, PI3K/Akt and/or MEK/ERK_1/2_ signaling pathways, but not CB2R.

#### 3.1.5. Retinal Ganglion Cells

It has been reported that WIN55,212-2, AEA, the selective CB1R agonist arachidonoyl-2-chloroethylamide (ACEA), and the CB2R agonist CB65 inhibit *I*_K_ via the tetraethylammonium (TEA)-sensitive K(+) current component in rat RGCs. These effects could not be reversed either by the CB1R inverse agonists AM251/SR141716A or by the CB2R inverse agonist AM630 [80] although both CB1Rs and CB2Rs are present on ganglion cells. The authors suggest that eCBs modulate potassium channels in rat RGCs in a receptor-independent manner, as demonstrated in other cells [81, 82]. An earlier* in vitro* study showed a partial inhibition of high-voltage activated Ca^2+^ channels with WIN55,212-2 that could be reversed by both SR141716A and AM281 in cultured rat RGCs [83]. A recent study showed that the agonist WIN55,212-2 caused a significant reversible reduction in the frequency of spontaneous postsynaptic currents (SPSCs) in RGCs of adult and young mice [84]. This effect was caused by presynaptic binding to cannabinoid receptors, as it did not alter the kinetics of SPSCs. The authors also found that the release probability of GABA and glutamate was significantly reduced by the agonist. As the largest reduction of the frequency of both GABAergic and glutamatergic SPSCs was observed in young mice, this suggests that the eCB system might play a role in the developmental maturation of synaptic circuits.

In addition to their effect at the level of ionic channels, eCBs, via CB1R, have neuroprotective properties. In fact, blockade of FAAH produces neuroprotective effects on RGCs in a rat model of optic nerve axotomy through a CB1R-mediated mechanism, which was gradually lost in aging animals [85]. Neuroprotective properties on RGCs were also reported with the administration of WIN55,212-2 in a rat model of acute rise of intraocular pressure induced ischemia [86]. Pinar-Sueiro et al. proposed that the neuroprotective effect of WIN55,212-2 was mediated by an inhibition of glutamatergic excitotoxicity, TNF-*α* release, and iNOS expression. But it is not confirmed experimentally. Thus, cannabinoid agonists, such as WIN55,212-2, could be relevant targets to prevent degeneration of RGC cells for intraocular pressure induced ischemia [85]. Overall, these neuroprotective effects by cannabinoid-mediated mechanisms are in accordance with a previous study reporting an increase in FAAH activity and a downregulation of AEA, CB1R, and TRPV1 following high intraocular pressure induced ischemia. This effect could be prevented by the administration of the FAAH inhibitor URB597 [26]. Furthermore, systemic administration of URB597 or intravitreal injection of methanandamide, a stable analogue of AEA, reduced cell loss in the GCL [26]. Furthermore, the neuroprotective effects mediated by cannabinoid ligands in the retina might be mediated by CB2R as well. Indeed, CB2R activation results in immunomodulation and neuroprotection in models of brain injury by altering infiltrating macrophages and activated resident microglia [87–89]. Additional studies are needed to assess the role of CB2R in retinal neuroprotection. Very little is known about the effects of other alternative cannabinoid receptors on RGC function. Future studies would need to point out if GPR55 or GPR18 activation affects RGCs.

Various TRP channels can be activated by tactile and pressure stimuli, including TRPV channels [90–93]. In RGCs, TRPV1 is activated by hydrostatic pressures in isolated rat cells* in vitro*. This activation causes an increase in intracellular Ca^2+^ and leads to cell apoptosis and could be partially prevented by using a nonspecific antagonist, iodoresiniferatoxin [42]. Ligands acting on the orthosteric site of the receptor, such as capsaicin, could also cause cell death. Interestingly, TRPV1 activity on microglia cells in the retina can have an inverse effect and prevent cell apoptosis when hydrostatic pressure is applied [94]. It would thus be appealing to verify if 2-AG, AEA, and N-arachidonoyl dopamine, all agonists of TRPV1, could act on RGCs via TRPV1 and mediate effects previously attributed to classical CBRs. Moreover, it would be interesting to validate if both receptors could act in synergy and lead to an increase in intracellular calcium inside these cells.

### 3.2. Cannabinoid Function in the Primary Visual Cortex

So far, few studies have looked at the effects of eCBs on central visual areas processing. CB1R activation alters spontaneous and visual activity in the rat dLGN, increasing the spontaneous bursting and oscillatory activity [95, 96]. For 28% of the geniculate cells, the injection of various agonists, such as AEA, 2-AG, and O2545, a potent water-soluble synthetic cannabinoid, increased the visual responses, while for the remaining 72%, decreased visual discharges were observed. These effects could be blocked by AM251. These studies suggest that CB1R acts as a dynamic modulator of visual information being sent to V1. In layer 2/3 of V1, eCBs play a crucial role in the maturation of GABAergic release. The developmental maturation of GABAergic release between eye opening and puberty can be affected in dark-reared mice and this effect can be mimicked by CB1R agonists and blocked by antagonists and was similar in* cnr1*-KO mice [97, 98]. GABAergic synapses in layers 2/3 and 5 have also been shown to not mature normally in* cnr1*-KO animals. These results suggest that visually stimulated endocannabinoid-mediated long-term depression of GABAergic neurotransmission (iLTD) happens in the extragranular layer of the mouse visual cortex [99]. In another report, pharmacological blockade of cannabinoid receptors prevents the ocular dominance shift in layers II/III of V1, leaving plasticity intact in layer IV of juvenile mouse [100]. Moreover, the application of cannabinoid agonists leads to an increase in the amplitude and frequency of spontaneous inhibitory postsynaptic currents and miniature inhibitory postsynaptic currents in mouse V1 [101]. The systemic administration of a cannabinoid agonist can also modify the encoding of visual stimuli, primarily by delaying and broadening the temporal response functions of V1 and V2 neurons in macaque monkeys [102]. Finally, more studies are needed to assess the impact of CB2R function in the primary visual cortex. However, based on the impact of CB2R on retinal function, it is conceivable that CB2R could mediate a large variety of effects in the primary visual cortex.

## 4. Endocannabinoid Expression and Function during Retinal Development

As shown in other systems, eCBs are well known modulators of synaptic transmission and neuronal plasticity, mostly via presynaptic inhibitory mechanisms. The impact of the eCB system during CNS development has been documented in the last decade. The eCB system regulates the proliferation, migration, specification, and survival of neural progenitors [103], dictates the differentiation of neurons, and controls the establishment of synaptic connections [104] (for review, see [105]). The importance of eCBs during neuronal development is confirmed by the demonstration that maternal marijuana smoking or cannabinoid consumption during pregnancy causes cognitive, motor, and social deficits [106–108] (see [109] for review). In addition, 2-AG levels in the CNS progressively increase during embryonic development and then peak just after birth [110, 111]. However, eCB-mediated changes in developmental processes are not only limited to the higher brain structures but also can affect the development of the retina.

### 4.1. Retinal Endocannabinoid Expression

CB1R mRNA is expressed in the rat retina as early as embryonic age 13 (E13), which is a good indicator of its possible developmental implication [27]. By E15 to E17, the GCL expresses CB1R mRNA and, from E20-E21, it is present in the GCL as well as in an unspecified cell layer that appears to be the INL [27]. The CB1R is also expressed in the chick retinotectal system as soon as E4, with labeling in ganglion cells and the IPL and INL [112]. The CB1R, FAAH, DAGL*α*, and MAGL were also detected during postnatal development of the rat retina [50, 52, 56]. The CB1R is present in ganglion cells, amacrine cells, and horizontal and mitotic cells at P1 [56]. During retinal development, a transient expression of CB1R was reported in cones and bipolar cells. Moreover, FAAH was found at P1 in ganglion and cholinergic amacrine cells, and, in the course of development, it appeared in cones, horizontal and bipolar cells [52]. FAAH is transiently expressed in horizontal, cholinergic amacrine cells and cone bipolar cells, suggesting an important redistribution of the enzyme during postnatal retinal development. Additionally, DAGL*α* is expressed early in retinal development as its presence at P1 is observed in cone and rod photoreceptors, horizontal, amacrine, and ganglion cells [50]. MAGL expression is detected at low levels from P1 to P9 and then it gradually elevated onto adulthood [50]. MAGL is constantly found in amacrine and Müller cells from P11 onto the adult age. Overall, the expression of DAGL*α* combined with the absence of MAGL expression in early postnatal development of the retina suggests that 2-AG levels could be elevated and thus play an active role in retinal development. Despite an important body of literature currently available on the involvement of eCBs in developmental functions, only few studies focused on the impact of cannabinoids in retinal development (see Section 4.2). It is to be noted that retinas from* cnr1*-KO and* cnr2*-KO animals do not show obvious changes in retinal structures, as their thickness, distribution, and morphology were similar to wild-type animals [33]. For these reasons, it is too early to hypothesize on the role of eCBs in retinal development.

The TRPV1 receptor is also present in the rat retina from E19 onto adulthood [41]. From E19 to P5, the TRPV1 receptor is detected in the neuroblastic layer in the pigment epithelium and in a few small cell bodies in the GCL. From P15 onto adulthood, TRPV1 is also present in all cell layers with prominent labeling in the IPL and GCL. The precise cellular expression of TRPV1 in the retina is still incomplete. So far, TRPV1 has been located in RGC [42] and microglial cells [94].

No studies have yet reported the expression of GPR18 in the developing retina. Ongoing projects from our group have so far shown that GPR55 mRNA and protein are expressed in the retina of newly born mice, more specifically in RGCs [113].

### 4.2. Endocannabinoid System Function

Only two studies reported the functional impact of cannabinoids on retinal development. CB1R and CB2R specific agonists induce a collapse of the growth cone of RGC axon and decrease axon growth [114, 115]. Conversely, the application of CB1R and CB2R specific inverse agonists increases the growth cone surface area and the number of filopodia present on the growth cone and increases axon growth. The intraocular injection of CB1R and CB2R specific inverse agonists promotes retinal projection growth in the dorsal terminal nucleus. Moreover, the importance of GPR55 in neuronal development has recently been pointed out by the lipid-signaling of GPR55 and one of its ligands, lyso-phosphatidyl-*β*-D-glucoside, in glial-neuron communication in the developing spinal cord [116]. GPR55 also modulates the growth rate and the targets innervation of retinal projections during development. For instance, the application of GPR55 agonists increases the growth cone surface area and the number of filopodia on the growth cone and also increases the axon growth [113]. Furthermore,* gpr55* ^−/−^ mice revealed a decreased branching in the dorsal terminal nucleus and a lower level of eye-specific segregation of retinal projections in the superior colliculus and in the dLGN. Altogether, these studies identify a mechanism by which the eCB system modulates retinothalamic development and offer a potential model to explain why cannabinoid agonists affect CNS development.

## 5. Future Directions and Implications

As we have reported in this review, CB1R and CB2R are both present in various retinal tissue where they modulate, in the most part via retrograde signaling, neurotransmitter release and where they inhibit potassium and calcium currents. These effects can thus modulate visual activity as early as the retina level. More studies are obviously needed to assess to what extent a cannabinoid-mediated modulation in the retina could impact visual perception. This is especially true given that cannabinoid receptors are present in most retinal cell types. As cannabinoids are also present in the visual cortex (including areas beyond V1 and V2), as well as subcortical regions such as the LGN, it may well be that cannabinoids modulate visual perception at each level of visual system hierarchy.

The ability of the eCB system to induce various changes in plasticity in central visual areas is now well described (for review, see [2]), and some of these plastic changes could potentially be present in the retina. Furthermore, in the CNS, and as our group and others have previously demonstrated in the visual system, cannabinoids play an important role in neuronal development and axon guidance. It would now be of interest to see if a similar variation in the concentration of receptors, enzymatic machinery, and endogenous ligands occur during retinal development. Moreover, a better anatomical identification of the various cannabinoids synthesizing and degrading enzymes, novel eCBs, and other potential cannabinoid-like receptors such as TRPV1, GPR55, and GPR18 are also needed in order to draw a clearer picture of all the possible targets for various cannabinoid ligands in the retina and the CNS. New methods are also needed to better localize and find the rapid occurrence of the activation of CBRs. Finally, some mechanisms associated with the modulation of the eCB system in specific areas of the retina have neuroprotective potential and regulate apoptosis and could even potentially help prevent pathologies of the retina, such as glaucoma and AMD.

## 6. Conclusion

Endocannabinoids constitute one of the newest neuromodulators found in neural and nonneural tissues throughout the body. Their wide expression in the nervous system and peripheral organ systems highlights the range of their actions and their potential in therapeutic applications. Strong evidence now suggests a wide distribution of eCBs, receptors, and enzymatic machinery in key structures of the visual system, including a strong presence in the retina. Although no clear picture can ascertain the specific effects cannabinoids can have in the retina itself, or the visual system as a whole, various mechanisms in specific cellular structures of the retina have now been reported. The cannabinoid system also appears to have several roles in neuronal survival and apoptosis in the retina and could be linked with many other ocular disorders. However, their specific mechanisms in retinal development, neuroplasticity, and neuroprotection need to be more thoroughly investigated.

## Figures and Tables

**Table 1:** Endocannabinoid levels in the adult retina of various species.

Endocannabinoids	Concentration (pmol/g)	Species
2-arachidonoyl glycerol (2-AG)	2,970 1,393 1,600	Rat [17] Human [20] Bovine [25]

Anandamide (AEA)	Under detection level 20,000 36 64	Rat [17] Rat [26] Human [20] Bovine [25]

Palmitoylethanolamide (PEA)	130 200	Rat [17] Human [20]

Oleoylethanolamide (OEA)	55	Rat [17]

**Table 2:** Cannabinoid receptor type 1 protein distribution in the adult retina of various species.

Retinal cells	

Photoreceptors	Expression in the inner [17, 56] and outer segments [22] Strong labeling in the cone pedicles [17, 51, 53, 56, 78]

Horizontal cells	Expression in the membrane but not in dendrites [17, 51, 56]

Bipolar cells	Expression in the dendrites, cell body, and axons of rod bipolar cells [17, 51]

Amacrine cells	Expression in amacrine cells, including GABAergic amacrine cells [17, 51, 56, 65]

Inner plexiform layer	Unspecified expression in the IPL [17, 22, 53] Expression in the synapses of rod bipolar cells [51] Higher expression in the synapses of ON cone bipolar cells compared to OFF cone bipolar cells [78]

Ganglion cells	Expression in the cell body and fibers [17, 22, 53, 56, 83]

Müller cells	Absence of expression [17, 53, 56] Expression in the goldfish retina only [78]

**Table 3:** Cannabinoid receptor type 2 protein distribution in the adult retina.

Retinal cells	

Photoreceptors	Expression in the outer and inner segments of cones [33] Absence of expression in cone pedicles [33] Expression in the inner and outer segments and cell body of rods [33]

Horizontal cells	Expression at the membrane of the soma and in horizontal cells, dendrites [32, 33]

Bipolar cells	Expression in the membrane of the soma and axons of rod bipolar cells [33] Expression in the membrane of the soma of cone bipolar cells [33]

Amacrine cells	Expression in some subtypes [32, 33]

Ganglion cells	Expression in the soma [32, 33]

Müller cells	Absence of expression at the membrane of the soma, in Müller cells, inner and outer processes [33] Expression in Müller cells' processes in the vervet monkey only [34]
